# Letter to the Editor: Problems with studying community-level pesticide storage to prevent suicide

**DOI:** 10.1186/s13063-021-05052-8

**Published:** 2021-01-28

**Authors:** Aastha Sethi, Michael Eddleston

**Affiliations:** Centre for Pesticide Suicide Prevention, University of Edinburgh, QMRI, 47 Little France Crescent, Edinburgh, EH16 4TJ UK

An estimated 28.75% of the world’s suicides occur in India [1, 2], emphasising the need for evidence-based interventions for suicide prevention that are particular to India and South Asia. We therefore read with great interest Pathare and colleagues’ study protocol for a cluster-randomised controlled trial evaluating a programme of three interventions in Gujarat, India [3].

The approach includes a secondary school intervention to reduce suicidal ideation among adolescents, a community-level pesticide storage facility to reduce access to pesticides at moments of crisis, and training for community health workers in recognition, management, and referral of people at high suicide risk. Follow-up is planned for up to 12 months. We note that the design of the study, with the rate of suicide and attempted suicide by all means as the primary outcome, will not allow the effect of any one intervention to be tested or quantified.

Lethal pesticide self-poisoning is a particularly important means of suicide in India, because it is common (responsible for 30–40% of all suicides [4, 5]) and quite preventable. The role of the second intervention—pesticide storage—as a way of preventing suicides is therefore important to understand.

The World Health Organization (WHO) has supported several pilot studies to assess the feasibility of improved pesticide storage to prevent pesticide self-poisoning [6]. Both a modest-sized Chinese household storage study (10,000 locked boxes; 2 intervention townships, 8 control townships) [6] and a small Indian community storage study (2 community storage facilities; 2 intervention villages, 2 control villages) [7] showed a reduction in the number of cases in communities receiving boxes. However, both effects were likely due to markedly higher incidences in the intervention arms before the intervention (i.e. reversion to the mean) [6].

Compliance with pesticide storage interventions tend to fall steadily after the first year [8]. In the Chinese study, during the first year, only 30% of households locked the boxes; by the third year, this had fallen to just 4–13%, suggesting that many households contained pesticides that were not locked away [6]. Sri Lankan studies have shown that providing household containers actually increases risk, as farmers shift pesticide storage from the fields to household boxes [8, 9].

Community storage has its own issues. In the Indian case study, only 248 (34.4%) of 721 households owning land used the storage facility during the first 18 months. A key issue with community storage facilities is that they are commonly located centrally, meaning farmers must walk into the village to get their pesticides before walking out again, past their house, to get to their fields. This may explain in part the poor uptake of this intervention [6].

We have previously tested the effectiveness of improved household storage, recruiting more than 53,000 Sri Lankan households to a large cluster randomised control trial (RCT) [10]. Remarkably, this pre-existing evidence is not mentioned in Pathare et al.’s protocol. While use of a locked storage container to store pesticides was relatively high, at about 70% after 1 year and 53% after 3 years, this trial found absolutely no evidence that improved storage reduced self-poisoning, even within the first year. With much lower uptake of community storage facilities after just 1 year, it is hard to see a community approach offering clear benefit.

The WHO report recommended that studies should last ‘for at least three to five years to help determine whether or not substitution of suicide methods occurs when access to one method (i.e. pesticides) is limited’ [6]. The very short duration of Pathare et al.’s study will prevent collection of any data on the medium-term effects of the combined intervention.

Pesticide bans are one of the most cost-effective interventions for reducing self-poisoning deaths [11]. Pesticide poisoning simply needs to be made safe, by removing all highly hazardous pesticides from agricultural practice. Doing more studies of ‘safe storage’ distracts from the approach with most evidence. Bans have led to major reductions in total suicide numbers in other South Asian countries, by making pesticide self-poisoning much less likely to result in death [11, 12]. WHO now recommends pesticide bans as a highly cost-effective approach for suicide prevention [13]. It also places the responsibility for ensuring farmer and community safety on the pesticide industry and regulators, and not on the farmers—as is the unfortunate case with ‘safe storage’.

Fortunately, the Indian government is now moving in this direction with bans imposed on 18 pesticides in 2018–2020 (including methyl parathion, dichlorvos, phorate, and phosphamidon) which have killed hundreds of thousands of Indian citizens [14]. Recently, 27 more pesticides have been proposed for bans (importantly including monocrotophos and dimethoate) [15]. If implemented and enforced, these government actions will have a major impact on pesticide suicides, saving tens of thousands of lives—at a scale unimaginable with improved pesticide storage.

This trial will test whether the youth mental health intervention and community health workers reduces self-harm in the short term. However, the long-term cost-effectiveness of these approaches will not be assessed by this study. Further large-scale studies will be required to provide these data before they should be scaled up for national use. In the meantime, India’s recent pesticide bans will have major impacts on Indian suicides and are a cause for celebration.

## Data Availability

Not applicable

## Abbreviations

WHO: World Health Organization
RCT: Randomised control trial
